# Non-coding Transcripts from Enhancers: New Insights into Enhancer Activity and Gene Expression Regulation

**DOI:** 10.1016/j.gpb.2017.02.003

**Published:** 2017-06-06

**Authors:** Hongjun Chen, Guangshi Du, Xu Song, Ling Li

**Affiliations:** 1 Center for Functional Genomics and Bioinformatics, Key Laboratory of Bio-Resources and Eco-Environment of Ministry of Education, College of Life Science, Sichuan University, Chengdu 610064, China; 2 State Key Laboratory of Biotherapy, West China Hospital, Sichuan University, Chengdu 610041, China

**Keywords:** LncRNA, Enhancer transcription, eRNA, Enhancer activity, Gene expression

## Abstract

Long non-coding RNAs (lncRNAs) have gained widespread interest in the past decade owing to their enormous amount and surprising functions implicated in a variety of biological processes. Some lncRNAs exert function as enhancers, *i.e.*, activating gene transcription by serving as the *cis*-regulatory molecules. Furthermore, recent studies have demonstrated that many enhancer elements can be transcribed and produce RNA molecules, which are termed as enhancer RNAs (**eRNAs**). The eRNAs are not merely the by-product of the **enhancer transcription**. In fact, many of them directly exert or regulate **enhancer activity** in gene activation through diverse mechanisms. Here, we provide an overview of enhancer activity, transcription of enhancer itself, characteristics of eRNAs, as well as their roles in regulating enhancer activity and **gene expression**.

## Introduction

Transcripts that are more than 200 nucleotides in length and lack the evident protein-coding potentiality are referred to as long non-coding RNAs (lncRNAs). lncRNAs not only contain functionally redundant sequences, but also exhibit low sequence conservation, which increases the complexity of their biological functions [1]. lncRNAs exert their functions through diverse mechanisms, including interaction with genomic DNA, proteins, mRNA, and other categories of ncRNAs, consequently regulating gene expression at multiple levels [2], [3], [4], [5], [6], [7], [8]. Recent studies have demonstrated that lncRNA-mediated regulation of gene expression is involved in embryogenesis, development, differentiation, and disease progression [9], [10], [11]. Therefore, lncRNAs have been thought to participate in the construction of organismal regulatory network by adding different layers to control gene expression.

Enhancers are a set of DNA elements that were initially revealed to positively modulate the transcription of nearby genes in an orientation-independent manner [12]. Subsequent studies further demonstrate that these elements possess the ability to orchestrate temporal and tissue-specific gene expression [13], [14]. Several effector models, such as “looping”, “tracking”, and “oozing”, have been proposed to explain how enhancers exert their functions [15]. While most findings appear to favor these models, the underlying molecular details remain largely unknown. Recently, a novel class of enhancer-transcribed ncRNAs, referred to as enhancer RNAs (eRNAs), have been uncovered [16], [17], [18]. eRNAs are 0.5–5 kb in length and therefore arbitrarily classified into lncRNAs [19]. The discovery of eRNAs, as well as their “emerging” ability to affect enhancer activity, has provided new insights into the enhancer action (Table 1).

**Table 1:** **The main timeline of eRNA studies**

**Year**	**Brief description and significance**	**Refs.**
1990, 1992	The early studies demonstrating that transcripts can be produced from enhancer regions	[20], [21]

2010	The first paper proposing the notion of eRNAs	[16]

2010, 2012	Genome-wide analysis suggesting that enhancers may be generally transcribed	[17], [18]

2013, 2014	Increasing evidence showing that eRNAs play an important role in regulating gene transcription via diverse mechanisms	[22], [23], [24], [25], [26], [27]

2014	*In vivo* study confirming that many eRNAs are expressed in a tissue-specific manner	[28]

2014	Study revealing a comprehensive transcriptomic profiling of eRNAs in humans	[29]

2015	Study illuminating the role of RNA exosome in controlling eRNA degradation	[30]

2016	Study showing that some eRNAs marked with m^5^C are responsible for metabolic stress	[31]


*Note*: This collection does not contain all the studies on eRNAs and only some representative articles are listed.

## Enhancer activity in gene regulation

Advances in genome-wide analysis technologies make it possible to investigate the chromatin features of enhancers. Using chromatin immunoprecipitation coupled with deep sequencing (ChIP-seq), it has been shown that enhancers with high activity usually display a low enrichment of H3K4me3, an epigenetic modification generally found at the promoter region of active genes [32], [33], [34]. As a matter of fact, the activated enhancers are specifically marked by H3K27ac, whereas the poised enhancers are generally characterized by the absence of H3K27ac and the enrichment of H3K27me3 and/or H3K9me3 [35]. A large number of enhancers with cell-type specificity have been found to share these epigenetic features [35]. Furthermore, the status of the poised enhancers could be reversed when the epigenetic modification of H3K27me3 is replaced by H3K27ac [35]. Interestingly, some transcription factors (TFs), including p300/CBP, are found to occupy the enhancer element, leading to an open chromatin conformation that confers high sensitivity of enhancers to DNase I [36], [37].

Some models, including “looping”, “tracking”, and “oozing”, have been proposed to explain how enhancers function. In the “tracking” model, enhancer is proposed to diffuse in one dimension along the chromatin to seek a promoter [38], whereas the “oozing” model presumes that a complex resides at the enhancer and then polymerizes alongside the chromatin bi-directionally until it hits a promoter [38], [39], [40]. The predominant one, however, is the “looping” model, which involves the loop formation between the promoter and the enhancer [41], [42]. Thanks to chromosome conformation capture (3C) technology and its high-throughput derivatives, this model has been supported by several studies aiming to reveal chromatin architecture [43], [44], [45], [46]. In accordance with the “looping” model, some complexes associated with chromatin architecture formation have been identified. For instance, mediator and cohesin are reported to co-occupy the enhancer and promoter, thus guiding the formation of chromatin loop [41], [42].

## Enhancers produce non-coding transcripts

In addition to TFs, RNA polymerase II (RNAPII) has also been found to be localized at many enhancers. In mouse cortical neurons, a large number of neuronal activity-controlled enhancers are recognized by the general transcriptional co-activator CREB binding protein (CBP). Upon KCl stimulation, CBP at enhancers recruits RNAPII and switches on the transcription [16]. In lipopolysaccharide (LPS)-stimulated mouse macrophages, the occupancy profile of the enhancer-related chromatin signature H3K4me1 indicated that 70% of extragenic transcription sites overlap enhancer elements. Further analysis using qRT-PCR showed that 96 out of 100 RNAPII-binding enhancers examined produce detectable transcripts [17]. Taken together, these studies provide strong evidence for the transcriptional potentiality of the enhancers.

Global nuclear run-on followed by high-throughput sequencing (GRO-seq) has been applied extensively to map nascent RNA across genome. Using this approach, it has been reported that enhancers recognized by androgen receptor (AR) are able to serve as transcription template to produce eRNAs during the reprogramming of hormonal response [47]. In macrophages, Rev-Erbs (Rev-Erb-α and Rev-Erb-β) and Kdo2-lipid A (KLA)-stimulated toll-like receptor 4 (TLR4) are well-characterized nuclear receptors that operate through impacting the enhancer activity [22], [48]. A number of Rev-Erb-binding enhancers display the active chromatin features, namely, the presence of H3K4me1 and the absence of H3K4me3 [22]. Additional GRO-seq-derived analysis has also shown that most of these enhancers undergo bidirectional transcription [22]. In the case of TLR4-regulated enhancer activity, GRO-seq analysis reveals widespread enhancer transcription in mouse macrophages upon the treatment with the TLR4 agonist KLA [49].

p53 is a core tumor suppressor that regulates the genes associated with cell proliferation and survival through recognizing and binding to the regulatory regions of transcription units [50]. Many of the p53-binding genomic regions share the enhancer hallmarks and produce non-coding transcripts in a p53-dependent manner [23]. Similarly, two independent groups have discovered that, in breast cancer cells, 17β-oestradiol (E2)-bound estrogen receptor α (ERα) binds to thousands of enhancers and causes enhancer transcription [24], [51]. In addition, activation of the transcription factor forkhead box O3 (FOXO3), which is associated with human longevity, has also been reported to potentiate production of non-coding transcripts from enhancers [52].

The β-globin locus control region (LCR) regulates transcription of the globin genes 10–50 kb away during erythroid cell differentiation [53]. Unexpectedly, the hypersensitive site 2 (HS2) enhancer in the β-globin LCR is found to undergo autonomous transcription in K562 cells, giving rise to several non-coding, intergenic RNAs [54]. In murine T cell populations, 7 DNase I-hypersensitive sites (DHSs) have been identified in *IL-10*, a key gene involved in suppressing cell-mediated immunity and necessary for the development of several T-regulatory cell populations. Among them, 5 DHSs act as enhancers and are transcribed to produce intergenic RNAs upon stimulus [55]. Differentiation of skeletal muscle is carried out by myogenic regulatory factors that include MyoD and MyoG [56], [57]. Interestingly, MyoD and MyoG occupy thousands of extragenic regions, which show enhancer features and are transcribed to produce non-coding transcripts [25]. More recently, Pulakanti et al. have reported that, in mouse embryonic stem cells (ESCs), numerous enhancers linked to pluripotency-associated genes are transcribed [58]. Collectively, the discovery of a large scale of non-coding transcripts named eRNAs indicates that enhancer transcription may be a common event in a variety of biological systems.

## eRNAs share some properties with lncRNAs

**Figure 1:**
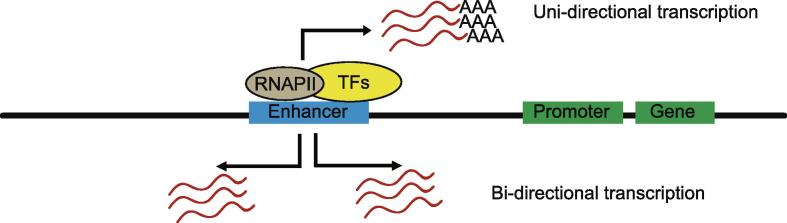
**Distinct types of eRNAs** Uni-directional and polyadenylated eRNAs are shown in the upper part of the diagram, whereas bi-directional and non-polyadenylated eRNAs are shown in the lower part of the diagram. RNAPII, RNA polymerase II; TF, transcription factor.

Besides the length and protein-coding potentiality, many eRNAs share some other properties with lncRNAs. Like the polyadenylated lncRNAs, most eRNAs are transcribed by RNAPII and retained in the nucleus [19]. In addition to the single-stranded form, some lncRNAs are found to exist as double-stranded molecules. A notable example is that Alu repeats are bidirectionally transcribed to facilitate formation of the RNA duplex [59]. Similarly, while certain enhancers can be transcribed uni-directionally, transcription of RNAPII-controlled enhancers is usually bi-directional, emanating both the sense and antisense transcripts [16], [24] (Figure 1). Interestingly, it has been reported that many intragenic enhancers serve as alternative promoters to generate a set of spliced, multi-exonic, and polyadenylated RNAs, termed as meRNAs [60]. Distinct from the eRNAs discussed above that are expressed at a low level, meRNAs are highly abundant in specific cell types. However, the detailed functions of these meRNAs await further interrogation.

## Functionality of eRNAs and lncRNAs in enhancer activity

Functions of eRNAs have been associated with enhancer activity. eRNA production from p53-bound enhancer regions (p53BERs) is p53-dependent and required for the p53-dependent activation of gene expression [23]. p53 activated by ionizing radiation induces eRNA production by increasing p53 binding to p53BERs. Moreover, although p53 is accumulated upon ionizing radiation, small interfering RNA (siRNA)-mediated knockdown of these eRNAs inhibits induction of the nearby p53 target genes [23]. Similarly, depletion of the eRNAs arising from ERα-binding sites following estradiol (E2) stimulation results in diminished transcription of the neighboring genes in human breast cancer cells [24].

Distinct from p53 and ERα, which induce eRNA transcription, the enhancer-binding nuclear receptor Rev-Erbs act conversely to inhibit enhancer transcription [22]. The Rev-Erb-controlled eRNAs are also involved in regulating enhancer activity and expression of their neighboring genes [22]. Given their specific expression profile in macrophage lineage, eRNAs may participate in the construction of the macrophage-specific gene regulatory network [22].

In the myogenic gene regulatory network, the core enhancer (CE) and two distal regulatory regions (DRRs) of *myoD1* are transcribed to eRNAs, namely ^CE^RNA and ^DRR^RNA [25]. After impeding the myogenic differentiation program, depletion of ^CE^RNA and ^DRR^RNA impairs the boost of *myoD* and *myoG* expression, respectively [25]. The lncRNA *Evf-2* is derived from the enhancer of *Dlx-5/6* upon sonic hedgehog (Shh) induction, coincident with *Dlx-5* and *Dlx-6* activation [61]. The enhancer activity is abolished upon *Evf-2* depletion, whereas enforced *Evf-2* expression promotes the enhancer activity, revealing the requirement of *Evf-2* for the enhancer activity. Taken together, these studies strongly point to eRNAs as the key players behind the DNA elements, enhancers, in regulation of gene transcription.

Like eRNAs, some lncRNAs derived from genomic regions other than enhancer also function in activating nearby gene via their “enhancer” function, which are named as eRNA-like lncRNAs. For example, *HOXA distal transcript antisense RNA* (*HOTTIP*), a long intergenic noncoding RNA (lincRNA) transcribed from the 5′ tip of the *HOXA* locus, is able to coordinate the activation of several 5′ *HOXA* genes in human anatomically distal cells [62]. Similarly, the lncRNA *Nettoie Salmonella pas Theiler’s* (NeST) is shown to exert enhancer-like function to activate the neighboring *interferon-γ* locus, thereby contributing to host susceptibility to microbial pathogens [63]. Moreover, some other lncRNAs termed as ncRNA-activating (ncRNA-a) have been discovered in many cell types, and they also participate in the transcriptional activation of neighboring protein-coding genes [64].

## The mechanisms underlying eRNA action

**Figure 2:**
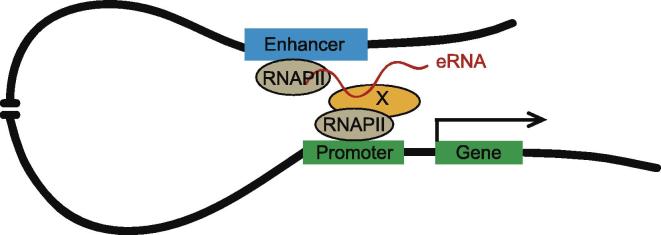
**Schematic model depicting how eRNAs work** The transcribed eRNAs interact with RNAPII and proteins (X), thus facilitating promoter–enhancer looping formation and then enhancing target gene transcription. RNAPII, RNA polymerase II; eRNA, enhancer RNA.

The eRNA-like lncRNAs exert transcription-promoting activity via diverse mechanisms. For example, ncRNA-a lncRNAs are proposed to work by facilitating long-range chromatin looping formation that alters chromosomal spatial structure [64]. Similar to ncRNA-a lncRNAs, most eRNAs exert enhancer function by mediating formation of the local promoter–enhancer looping (Figure 2). Using an RNA tethering reporter assay, studies on the eRNAs involved in p53BER regulation reveal p53BER interaction with several distant protein-coding genes, which is necessary for the activation of these p53 target genes [23]. Intriguingly, the long-range interactions between the promoter and enhancer are p53-independent, implying that eRNAs produced from p53BERs may act on pre-established chromatin conformations. Nevertheless, other factors involved in this process remain to be identified.

A more precise effector model for eRNAs has been proposed in a subsequent study by investigating roles of the E2-induced eRNAs in controlling the neighboring genes in MCF-7 cells [24]. Consistent with the results from tethering reporter assay, eRNA depletion mediated by siRNA or locked nucleic acid antisense oligos (LNAs) leads to reduced transcription of the adjacent coding genes, indicating that eRNAs *per se* are necessity for induction of neighboring genes, but not by-products of enhancer activation. Despite lack of effect on the binding of ERα to enhancer elements, eRNA depletion leads to substantial alteration in the specific promoter–enhancer interactions. Moreover, it has been shown that cohesin is recruited to the interrogated enhancers upon E2 treatment [65]. Notably, cohesin subunits can bind to eRNAs, and eRNA depletion decreases cohesion occupancy at enhancers, suggesting that eRNAs might act as “guiding” molecules to recruit the functional complexes. The E2-induced eRNA transcription has also been investigated in another study. Hah and colleagues indicate that eRNA repression caused by the transcriptional elongation inhibitor flavopiridol do not alter the specific promoter–enhancer interactions [51]. This seems to be in contrast with the effect of siRNA- or LNA-mediated eRNA depletion described above. Given that flavopiridol also represses the expression of protein-coding genes, it is speculated that the initial synthesis of eRNAs may be sufficient for chromatin looping establishment [66]. Therefore, eRNAs may guide gene activation in *cis* via recruiting chromatin modifiers and affecting chromosome conformation.

^CE^RNA and ^DRR^RNA derived from *myoD1* locus function through more complicated patterns [25]. Depletion of ^CE^RNA, but not that of ^DRR^RNA, interferes with the expression of neighboring gene *myoD*, indicating that ^CE^RNA is directly associated with enhancer-mediated *myoD* activation and exerts regulatory effect in *cis*. Although ^DRR^RNA depletion has little impact on *myoD* expression, deletion of the DRR DNA element is shown to reduce *myoD* expression in all myogenic lineages [67]. Consistent with the essential role of DRR in the early myogenic differentiation program, depletion of ^DRR^RNA abolishes the activation of myoD target genes (*e.g.*, *myoG* and *myh*) and hinders the myogenic differentiation program severely [25]. Of critical importance, overexpression of the 1.2 kb and 2.0 kb fragments of ^DRR^RNA can activate *myoG* expression and the myogenic gene regulatory network but has little effect on *myoD* transcription, suggesting that ^DRR^RNA exerts its function of gene activation in *trans* and in a *myoD*-independent manner. Although these two eRNAs, ^CE^RNA and ^DRR^RNA, function in *cis* and in *trans*, respectively, further experiments demonstrate that both of them promote chromatin accessibility and RNAPII assembly at specific loci to activate the corresponding target genes. Another example of eRNA that act in *trans* is *Evf-2*, since its enhancer activity could be promoted by ectopic overexpression of full-length *Evf-2* or its 5′ fragment [61]. Taken together, these studies reinforce the notion that eRNAs can act in *cis* or in *trans* to alter chromatin architecture.

It has also been reported that the process of enhancer transcription, in addition to the resultant eRNA transcripts, can mediate the enhancer activity. In the case of TLR4 induction, deposition of H3K4 methylation is established upon enhancer transcription [49]. Inhibition of eRNA elongation is correlated with a reduction in the deposition of local H3K4me2. However, LNA-mediated eRNA depletion exhibits minimal effect on the H3K4me2 deposition. These data manifest that enhancer transcription, but not the eRNA molecule itself, is required for the H3K4me2 deposition. Similarly, another study also confirms that enhancer activation in AR reprogramming relies on the process of enhancer transcription, and the produced eRNAs might only serve as “signal” molecules to indicate the active status of enhancers [47].

## Perspectives

Enhancers were discovered about thirty years ago, and their “classic” functions in activating gene expression have been well documented. Discovery of enhancer transcription and the resultant eRNAs provides new insight into the enhancer functions. Moreover, several groups have identified a set of super-enhancers that comprise multiple transcriptional enhancers, and found that these super-enhancers are associated with cell differentiation and diseases [68], [69], [70]. We are only beginning to understand the real realm of eRNAs. While several effector models have been proposed to explain how eRNAs exert their functions, the detailed molecular mechanisms through which enhancers become activated as transcription units remain largely mysterious. For example, what is the mechanism underlying expression and regulation of eRNAs? Is the transcription apparatus on enhancers identical to that on promoters? What are the *cis*-acting elements and *trans*-acting factors that determine the initiation, elongation, and termination of eRNA transcription? Enhancer aberration and eRNA-mediated gene activation have been implicated in diseases that include breast cancer [23], [24], [51]. Therefore, studies on the emerging eRNAs, especially their expression regulation, may provide new strategies for the therapy of diseases.

## Competing interests

The authors declare that they have no competing interests.

## References

[1] Ponting C.P., Oliver P.L., Reik W. (2009). Evolution and functions of long non-coding RNAs. Cell.

[2] Guttman M., Rinn J.L. (2012). Modular regulatory principles of large non-coding RNAs. Nature.

[3] Li L., Feng T., Lian Y., Zhang G., Garen A., Song X. (2009). Role of human non-coding RNAs in the control of tumorigenesis. Proc Natl Acad Sci U S A.

[4] Song X., Sui A., Garen A. (2004). Binding of mouse VL30 retrotransposon RNA to PSF protein induces genes repressed by PSF: effects on steroidogenesis and oncogenesis. Proc Natl Acad Sci U S A.

[5] Song X., Sun Y., Garen A. (2005). Roles of PSF protein and VL30 RNA in reversible gene regulation. Proc Natl Acad Sci U S A.

[6] Tripathi V., Ellis J.D., Shen Z., Song D.Y., Pan Q., Watt A.T. (2010). The nuclear-retained non-coding RNA *MALAT1* regulates alternative splicing by modulating SR splicing factor phosphorylation. Mol Cell.

[7] Wang G., Cui Y., Zhang G., Garen A., Song X. (2009). Regulation of proto-oncogene transcription, cell proliferation, and tumorigenesis in mice by PSF protein and a VL30 non-coding RNA. Proc Natl Acad Sci U S A.

[8] Yoon J.H., Abdelmohsen K., Srikantan S., Yang X., Martindale J.L., De S. (2012). LincRNA-p21 suppresses target mRNA translation. Mol Cell.

[9] Bernard D., Prasanth K.V., Tripathi V., Colasse S., Nakamura T., Xuan Z. (2010). A long nuclear-retained non-coding RNA regulates synaptogenesis by modulating gene expression. EMBO J.

[10] Eißmann M., Gutschner T., Hämmerle M., Günther S., Caudron-Herger M., Groß M. (2012). Loss of the abundant nuclear non-coding RNA *MALAT1* is compatible with life and development. RNA Biol.

[11] Kim K., Jutooru I., Chadalapaka G., Johnson G., Frank J., Burghardt R. (2013). *HOTAIR* is a negative prognostic factor and exhibits pro-oncogenic activity in pancreatic cancer. Oncogene.

[12] Banerji J., Rusconi S., Schaffner W. (1981). Expression of a β-globin gene is enhanced by remote SV40 DNA sequences. Cell.

[13] Ong C.T., Corces V.G. (2011). Enhancer function: new insights into the regulation of tissue-specific gene expression. Nature Rev Genet.

[14] Heintzman N.D., Hon G.C., Hawkins R.D., Kheradpour P., Stark A., Harp L.F. (2009). Histone modifications at human enhancers reflect global cell-type-specific gene expression. Nature.

[15] Bulger M., Groudine M. (2011). Functional and mechanistic diversity of distal transcription enhancers. Cell.

[16] Kim T.K., Hemberg M., Gray J.M., Costa A.M., Bear D.M., Wu J. (2010). Widespread transcription at neuronal activity-regulated enhancers. Nature.

[17] Santa F.D., Barozzi I., Mietton F., Ghisletti S., Polletti S., Tusi B.K. (2010). A large fraction of extragenic RNA pol II transcription sites overlap enhancers. PLoS Biol.

[18] Djebali S., Davis C.A., Merke A., Dobin A., Lassmann T., Mortazavi A. (2012). Landscape of transcription in human cells. Nature.

[19] Natoli G., Andrau J.C. (2012). Non-coding transcription at enhancers: general principles and functional models. Annu Rev Genet.

[20] Collis P., Antoniou M., Grosveld F. (1990). Definition of the minimal requirements within the human β-globin gene and the dominant control region for high level expression. EMBO J.

[21] Tuan D., Kong S., Hu K. (1992). Transcription of the hypersensitive site HS2 enhancer in erythroid cells. Proc Natl Acad Sci U S A.

[22] Lam M.T., Cho H., Lesch H.P., Gosselin D., Heinz S., Tanaka-Oishi Y. (2013). Rev-Erbs repress macrophage gene expression by inhibiting enhancer-directed transcription. Nature.

[23] Melo C.A., Drost J., Wijchers P.J., van de Werken H., de Wit E., Oude Vrielink J.A. (2013). eRNAs are required for p53-dependent enhancer activity and gene transcription. Mol Cell.

[24] Li W., Notani D., Ma Q., Tanasa B., Nunez E., Chen A.Y. (2013). Functional roles of enhancer RNAs for oestrogen-dependent transcriptional activation. Nature.

[25] Mousavi K., Zare H., Dell'orso S., Grontved L., Gutierrez-Cruz G., Derfoul A. (2013). eRNAs promote transcription by establishing chromatin accessibility at defined genomic loci. Mol Cell.

[26] Fang B., Everett L.J., Jager J., Briggs E., Armour S.M., Feng D. (2014). Circadian enhancers coordinate multiple phases of rhythmic gene transcription *in vivo*. Cell.

[27] Hsieh C.L., Fei T., Chen Y., Li T., Gao Y., Wang X. (2014). Enhancer RNAs participate in androgen receptor-driven looping that selectively enhances gene activation. Proc Natl Acad Sci U S A.

[28] Wu H., Nord A.S., Akiyama J.A., Shoukry M., Afzal V., Rubin E.M. (2014). Tissue-specific RNA expression marks distant-acting developmental enhancers. PLoS Genet.

[29] Andersson R., Gebhard C., Miguel-Escalada I., Hoof I., Bornholdt J., Boyd M. (2014). An atlas of active enhancers across human cell types and tissues. Nature.

[30] Pefanis E., Wang J., Rothschild G., Lim J., Kazadi D., Sun J. (2015). RNA exosome-regulated long non-coding RNA transcription controls super-enhancer activity. Cell.

[31] Aguilo F., Li S.D., Balasubramaniyan N., Sancho A., Benko S., Zhang F. (2016). Deposition of 5-methylcytosine on enhancer RNAs enables the coactivator function of PGC-1a. Cell Rep.

[32] Visel A., Blow M.J., Li Z., Zhang T., Akiyama J.A., Holt A. (2009). ChIP-seq accurately predicts tissue-specific activity of enhancers. Nature.

[33] Lee S., Lee D.K., Dou Y., Lee J., Lee B., Kwak E. (2006). Coactivator as a target gene specificity determinant for histone H3 lysine 4 methyltransferases. Proc Natl Acad Sci U S A.

[34] Heintzman N.D., Stuart R.K., Hon G., Fu Y., Ching C.W., Hawkins R.D. (2007). Distinct and predictive chromatin signatures of transcriptional promoters and enhancers in the human genome. Nat Genet.

[35] Creyghton M.P., Cheng A.W., Welstead G.G., Kooistra T., Carey B.W., Steine E.J. (2010). Histone H3K27ac separates active from poised enhancers and predicts developmental state. Proc Natl Acad Sci U S A.

[36] Gross D.S., Garrard W.T. (1988). Nuclease hypersensitive sites in chromatin. Annu Rev Biochem.

[37] Ghisletti S., Barozzi I., Mietton F., Polletti S., De Santa F., Venturini E. (2010). Identification and characterization of enhancers controlling the inflammatory gene expression program in macrophages. Immunity.

[38] Blackwood E.M., Kadonaga J.T. (1998). Going the distance: a current view of enhancer action. Science.

[39] Zhu X., Ling J., Zhang L., Pi W., Wu M., Tuan D. (2007). A facilitated tracking and transcription mechanism of long-range enhancer function. Nucleic Acids Res.

[40] Miele A., Dekker J. (2008). Long-range chromosomal interactions and gene regulation. Mol Biosyst.

[41] Nolis I.K., McKay D.J., Mantouvalou E., Lomvardas S., Merika M., Thanos D. (2009). Transcription factors mediate long-range enhancer-promoter interactions. Proc Natl Acad Sci U S A.

[42] Kagey M.H., Newman J.J., Bilodeau S., Zhan Y., Orlando D.A., van Berkum N.L. (2010). Mediator and cohesin connect gene expression and chromatin architecture. Nature.

[43] Miele A., Dekker J. (2009). Mapping *cis*- and *trans*- chromatin interaction networks using chromosome conformation capture (3C). Methods Mol Biol.

[44] Dekker J., Rippe K., Dekker M., Kleckner N. (2002). Capturing chromosome conformation. Science.

[45] Zhao Z., Tavoosidana G., Sjölinder M., Göndör A., Mariano P., Wang S. (2006). Circular chromosome conformation capture (4C) uncovers extensive networks of epigenetically regulated intra- and interchromosomal interactions. Nat Genet.

[46] Dostie J., Richmond T.A., Arnaout R.A., Selzer R.R., Lee W.L., Honan T.A. (2006). Chromosome conformation capture carbon copy (5C): a massively parallel solution for mapping interactions between genomic elements. Genome Res.

[47] Wang D., Garcia-Bassets I., Benner C., Li W., Su X., Zhou Y. (2011). Reprogramming transcription by distinct classes of enhancers functionally defined by eRNA. Nature.

[48] Raetz C.R., Garrett T.A., Reynolds C.M., Shaw W.A., Moore J.D., Smith D.C. (2006). Kdo2-lipid A of *Escherichia coli*, a defined endotoxin that activates macro phages via TLR-4. J Lipid Res.

[49] Kaikkonen M.U., Spann N.J., Heinz S., Romanoski C.E., Allison K.A., Stender J.D. (2013). Remodelling of the enhancer landscape during macrophage activation is coupled to enhancer transcription. Mol Cell.

[50] Laptenko O., Prives C. (2006). Transcriptional regulation by p53: one protein, many possibilities. Cell Death Differ.

[51] Hah N., Murakami S., Nagari A., Danko C.G., Kraus W.L. (2013). Enhancer transcripts mark active estrogen receptor binding sites. Genome Res.

[52] Eijkelenboom A., Mokry M., Smits L.M., Nieuwenhuis E.E., Burgering B.M. (2013). FOXO3 selectively amplifies enhancer activity to establish target gene regulation. Cell Rep.

[53] Tuan D., Solomon W., Li Q., London I.M. (1985). The “beta-like-globin” gene domain in human erythroid cells. Proc Natl Acad Sci U S A.

[54] Ling J., Baibakov B., Pi W., Emerson B.M., Tuan D. (2005). The HS2 enhancer of the beta-globin locus control region initiates synthesis of non-coding, polyadenylated RNAs independent of a *cis*-linked globin promoter. J Mol Biol.

[55] Jones E.A., Flavell R.A. (2005). Distal enhancer elements transcribe intergenic RNA in the IL-10 family gene cluster. J Immunol.

[56] Jin Y., Murakami N., Saito Y., Goto Y., Koishi K., Nonaka I. (2000). Expression of MyoD and myogenin in dystrophic mice, mdx and dy, during regeneration. Acta Neuropathol.

[57] Weis J. (1994). Jun, Fos, MyoD1, and myogenin proteins are increased in skeletal muscle fiber nuclei after denervation. Acta Neuropathol.

[58] Pulakanti K., Pinello L., Stelloh C., Blinka S., Allred J., Milanovich S. (2013). Enhancer transcribed RNAs arise from hypomethylated Tet-occupied genomic regions. . Epigenetics.

[59] Wang P., Yin S., Zhang Z., Xin D., Hu L., Kong X. (2008). Evidence for common short natural trans sense–antisense pairing between transcripts from protein coding genes. Genome Biol.

[60] Kowalczyk M.S., Hughes J.R., Garrick D., Lynch M.D., Sharpe J.A., Sloane-Stanley J.A. (2012). Intragenic enhancers act as alternative promoters. Mol Cell.

[61] Feng J., Bi C., Clark B.S., Mady R., Shah P., Kohtz J.D. (2006). The *Evf-2* noncoding RNA is transcribed from the Dlx-5/6 ultraconserved region and functions as a Dlx-2 transcriptional coactivator. Genes Dev.

[62] Wang K.C., Yang Y.W., Liu B., Sanyal A., Corces-Zimmerman R., Chen Y. (2011). A long noncoding RNA maintains active chromatin to coordinate homeotic gene expression. Nature.

[63] Gomez J.A., Wapinski O.L., Yang Y.W., Bureau J.F., Gopinath S., Monack D.M. (2013). The NeST long ncRNA controls microbial susceptibility and epigenetic activation of the interferon-γ locus. Cell.

[64] Lai F., Orom U.A., Cesaroni M., Beringer M., Taatjes D.J., Blobel G.A. (2013). Activating RNAs associate with mediator to enhance chromatin architecture and transcription. Nature.

[65] Li W., Notani D., Ma Q., Tanasa B., Nunez E., Chen A.Y. (2013). Functional importance of eRNAs for estrogen-dependent transcriptional activation events. Nature.

[66] Chao S.H., Price D.H. (2001). Flavopiridol inactivates P-TEFb and blocks most RNA polymerase II transcription *in vivo*. J Biol Chem.

[67] Chen J.C., Ramachandran R., Goldhamer D.J. (2002). Essential and redundant functions of the *MyoD* distal regulatory region revealed by targeted mutagenesis. Dev Biol.

[68] Whyte W.A., Orlando D.A., Hnisz D., Abraham B.J., Lin C.Y., Kagey M.H. (2013). Master transcription factors and mediator establish super-enhancers at key cell identity genes. Cell.

[69] Hnisz D., Abraham B.J., Lee T.I., Lau A., Saint-André V., Sigova A.A. (2013). Super-enhancers in the control of cell identity and disease. Cell.

[70] Lovén J., Hoke H.A., Lin C.Y., Lau A., Orlando D.A., Vakoc C.R. (2013). Selective inhibition of tumor oncogenes by disruption of super-enhancers. Cell.

